# Effectiveness of P-wave ECG index and left atrial appendage volume in predicting atrial fibrillation recurrence after first radiofrequency catheter ablation

**DOI:** 10.1186/s12872-021-01930-w

**Published:** 2021-04-06

**Authors:** Ruibin Li, Xiaohong Yang, Min Jia, Dong Wang, Xiaoran Cui, Long Bai, Lei Zhao, Jidong Zhang

**Affiliations:** Department of Cardiology, Second Hospital of Hebei Medical University, No. 215 West Heping Road, Shijiazhuang, 050000 Hebei China

**Keywords:** P-wave ECG index, Left atrial appendage volume, Radiofrequency catheter ablation of atrial fibrillation, Recurrence

## Abstract

**Background:**

The primary aim was to observe the predictive value of P-wave ECG index and left atrial appendage volume (LLAV) for atrial fibrillation recurrence after first radiofrequency catheter ablation.

**Methods:**

A total of 196 patients with paroxysmal atrial fibrillation were enrolled. The preoperative LLAV was measured by cardiac enhanced CT. The P-wave ECG index including minimum P-wave duration (P-min), maximum P-wave duration (P-max), mean P-wave duration (mPWD), P-wave dispersion (PWD), P-wave terminal force in lead V1 (PtfV1), PR interval prolongation, and interatrial block (IAB) were analyzed and recorded in 12-lead ECG of sinus rhythm.

**Results:**

According to the follow-up results, the patients were divided into two groups: the non-recurrence group and the recurrence group. P-min, PWD, P-max, PtfV1 ≥ 0.04 mV·s, PR interval prolongation, and the ratio of first and third-degree IAB in the recurrence group were higher than those in the non-recurrence group, with significant statistical differences (P < 0.05). Kaplan–Meier curve analysis was performed on time to atrial fibrillation recurrence after catheter ablation when PtfV1 ≥ 0.04 mv s by comparison between groups (Log Rank test: 2 = 4.739, P < 0.001). Kaplan–Meier curve analysis showed that the survival rate without recurrence of atrial fibrillation after catheter ablation was lower when the LLAV exceeded 8.0 mL (log-rank test P < 0.001).

**Conclusion:**

PWD, P-max, PtfV1, PR interval prolongation, first and third-degree IAB, and LLAV can effectively predict atrial fibrillation recurrence after radiofrequency catheter ablation. The combination might be a valid and alternative independent predictor of recurrence.

## Background

Atrial fibrillation is a common arrhythmia in clinical practice. Based on the duration of symptom, atrial fibrillation can be categorized into permanent, persistent and paroxysmal atrial fibrillation. Atrial fibrillation often causes several symptoms, including shortness of breath, chest discomfort, vertigo, palpitation, and other symptoms, with an increased risk of stroke and heart failure. The condition poses a serious threat and adverse impact on the quality of life and the lives of patients [1, 2]. Radiofrequency catheter ablation is commonly used to treat atrial fibrillation [3, 4], with a success rate as high as 60–90%. However, there is still a risk of recurrence for patients. It is always important for clinical treatment of disease to timely and accurately identify the predictors of recurrence with an attempt to reduce the risk of treatment and medical expenses of patients, hence improving the success rate of the operation. At present, few clinical indicators exist to predict the recurrence of paroxysmal atrial fibrillation after radiofrequency catheter ablation. Therefore, the current study was conducted to observe and explore the predictive effect and potential clinical value of P-wave ECG index and left atrial appendage volume for atrial fibrillation recurrence after first radiofrequency catheter ablation, with an attempt to offer evidence and insight for clinical practice.

## Methods

### Clinical background

Totally 196 patients with paroxysmal atrial fibrillation underwent the first successful radiofrequency catheter ablation of atrial fibrillation in our hospital between November 2017 to November 2018. They were followed up for over 12 months after discharge. The symptoms and ECG information of the patients were closely monitored. Recurrence was defined as at least one occurrence of rapid atrial arrhythmia for more than 30 s in 12 months after Coronary according to the follow-up results, and the patients were divided into two groups: the non-recurrence group (n = 155) and the recurrence group (n = 41). There were 109 males and 46 females, aged 48.97–65.09 years, with an average age of (55.87 ± 3.98) years in the non-recurrence group. The non-recurrence group included 27 males and 14 females, aged 47.027–65.13 years, with an average age of (55.28 ± 4.01) years. The sex and age of patients in the two groups were comparable (P > 0.05). To be included in the present study, the following inclusion criteria should be met: (1) patients with paroxysmal atrial fibrillation who were treated successfully after radiofrequency catheter ablation of atrial fibrillation for the first time; (2) patients whose left atrial thrombus was not found by 64-slice spiral CT before the operation, and the left atrium and left atrial auricle were well filled with a contrast agent, and the boundary of them could be distinguished by CT scan; (3) informed consent form was required before radiofrequency catheter ablation of atrial fibrillation; (4) clinical data were complete; (5) there were no serious postoperative complications. Exclusion criteria were as follows: (1) poor quality of cardiac enhanced CT images, unclear and unmeasurable volume boundaries of the left atrium and the left atrial appendage; (2) hyperthyroidism; (3) incomplete electrical isolation of the circumferential pulmonary vein; (4) cardiac function grade 4 (New York cardiac function class); (5) severe hepatorenal insufficiency; (6) patients who were complicated with valvular heart disease or congenital heart disease; (7) patients who were complicated with severe chronic obstructive pulmonary disease. (8) patients who were unconscious and mentally abnormal; (9) patients had surgically treated cerebral infarction or cerebral hemorrhage; (10) disagreement, loss to follow-up, or withdrawal of consent; (11) familial hypercholesterolemia.

### General clinical data and laboratory results were collected before operation

The basic clinical data of patients were collected through the electronic medical record system, including gender, age, BMI, basic heart disease, concomitant diseases, and the use of drugs for treatment. Fasting venous blood (3–5 mL) was taken before the operation, and the laboratory indexes such as *N*-terminal B-type natriuretic peptide precursor, uric acid, blood urea nitrogen, creatinine, C-reactive protein, and cardiac troponin I were detected after centrifugation. Cobase411 produced by Roche Co. was used to detect the precursor of *N*-terminal B-type brain natriuretic peptide and cardiac troponin I by electrochemiluminescence. C-reactive protein was detected by immune turbidimetry, and creatinine, blood urea nitrogen, and uric acid were detected through the enzymatic method with C8000 (Abbott Co.).

### Determination of ECG indexes

**Fig. 1:**
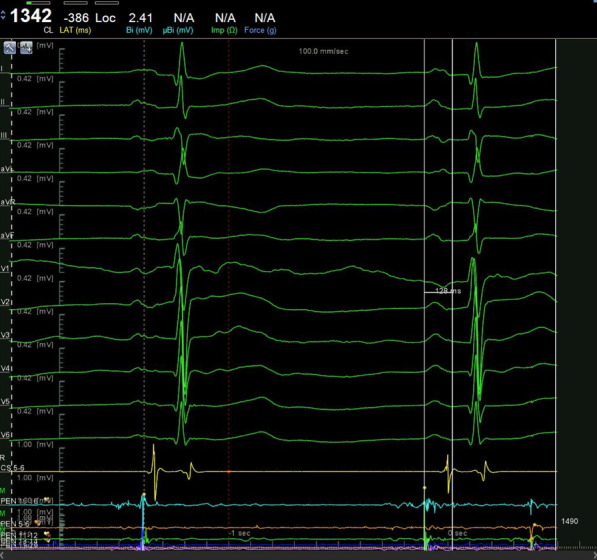
Pmax in the 12-lead ECG. The maximum width of P wave is 128 ms

**Fig. 2:**
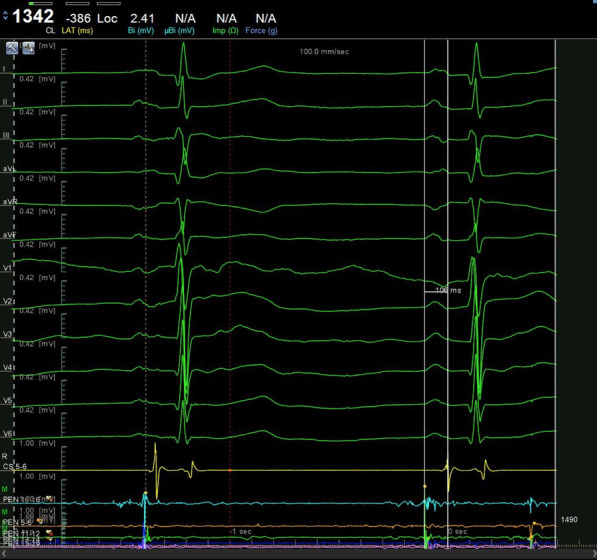
Pmin in the 12-lead ECG. The minimum width of P wave is 106 ms

**Fig. 3:**
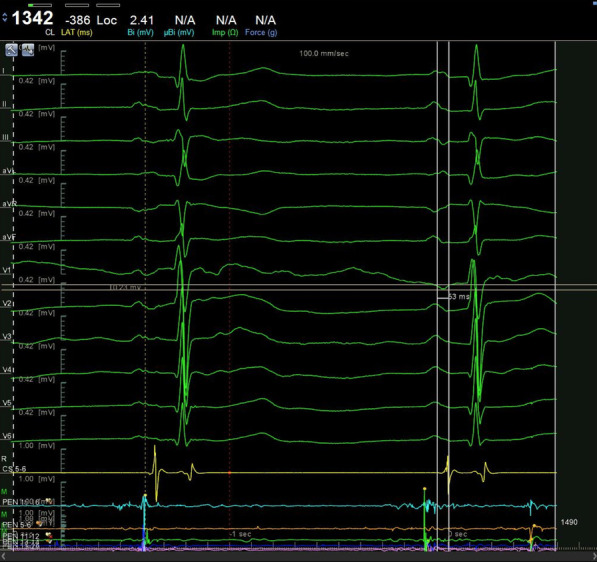
P_t_fV_1_ = duration (s) × P-wave terminal amplitude (mV). The P duration was 0.053 s, and P-wave terminal amplitude was 0.23 mV

The 12-lead ECG of sinus rhythm was recorded and analyzed in all patients before operation. P-wave dispersion (PWD), minimum P-wave duration (P-min), maximum P-wave duration (P-max), and P-wave terminal force in lead V1 (PtfV1) were measured and the mean values were calculated (Figs. 1, 2 and 3). PR is the distance from the starting point of the P-wave to the starting point of the QRS wave. PWD = P-max (all leads)-P-min (all leads); PtfV1 = duration (s) × P-wave terminal amplitude (mV). Interatrial block (IAB): first degree IAB (also known as partial IAB, pIAB): The P wave electrocardiogram axis is normal, and excitation is conducted from the right atrium to the left atrium through the normal pathway. There is a conduction delay in the Bachmann bundle, PWD > 120 ms. P wave notch are found in leads I, II, III, and sometimes P wave of V4-V6 leads can also show notches.

### Determination of the left atrial appendage and left atrial volume

**Fig. 4:**
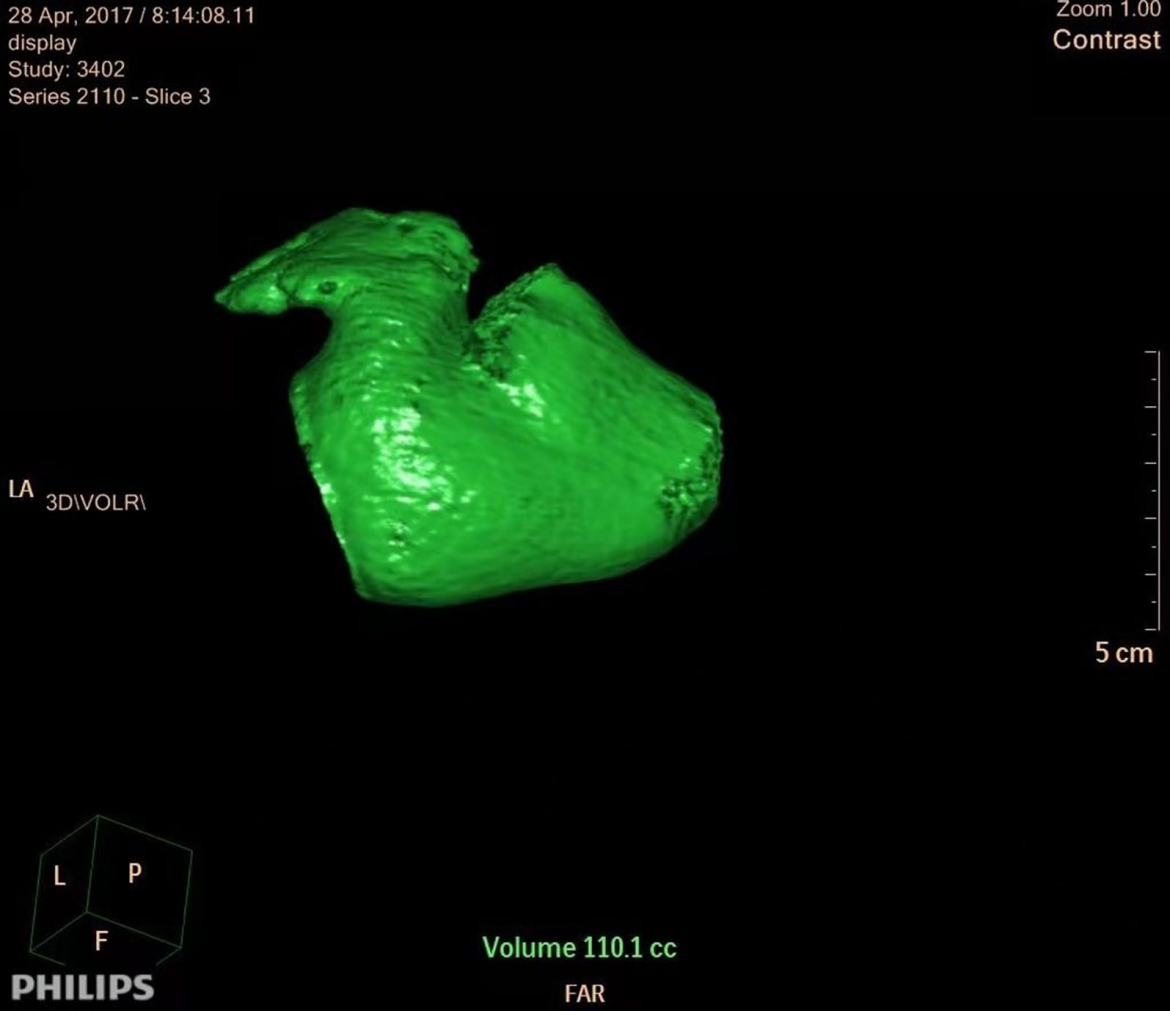
Three-dimensional volume of the left atrium in CT

**Fig. 5:**
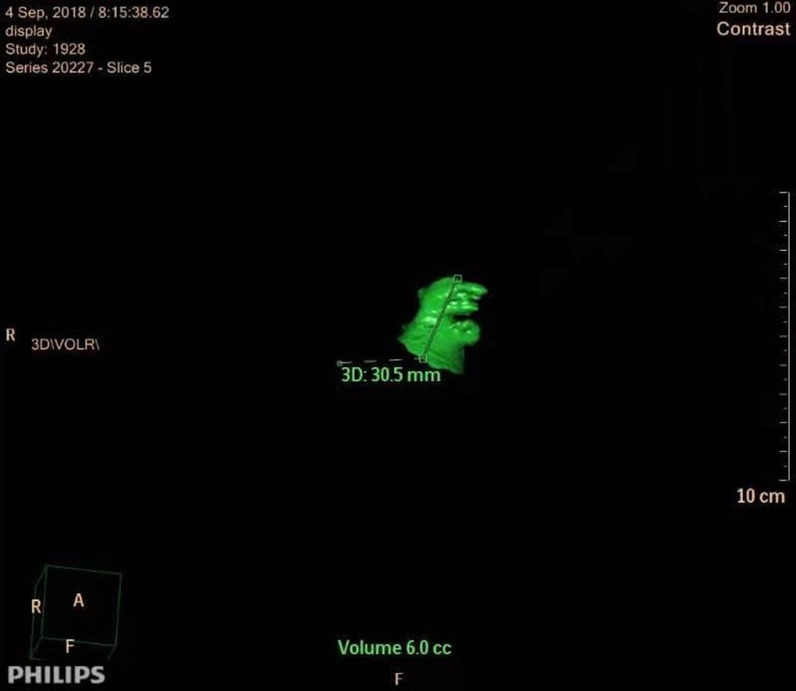
Three-dimensional volume of the left atrial appendage in CT

The results of preoperative cardiac enhanced CT in all patients were recorded and analyzed, and the volume of the left atrial appendage was measured accurately. CT scan was done by 64-row spiral CT (Siemens). Patients were injected with 90 mL ioproamine from the anterior elbow vein to maintain the speed of 4–5 mL/s, followed by 40 mL 0.9% saline injected at 4–5 mL/s. The retrospective ECG current regulation technique was used to collect image data, and the maximum tube current (450–500 mA) was used in the systole of the cardiac cycle, while in other periods, the appropriate current could be selected to reduce the current properly. The images were analyzed by Intuition platform software (TeraRecon Co., USA). The largest left atrium and left atrial appendage volume at the end of the systole was measured before mitral valve opening, so the left ventricle volume was the smallest and the left atrium volume was the largest. The left atrium and left atrial appendage margin were drawn manually on the axial section, and the left atrium and left atrial appendage orifice were accurately distinguished by the orthogonal view, in which the left atrial appendage cavity, including the left atrial appendage muscle trabecula, and the three-dimensional volume of the left atrial appendage and the left atrium were calculated by Simpson method (Figs. 4, 5). In addition, the diameter, volume of the left atrium and left ventricular ejection fraction were measured by transesophageal echocardiography. To avoid the bias of the abovementioned measurement, two deputy chief physicians in our hospital examined the cardiac enhanced CT scans of the patients 3 times each time, and the average value was taken, while ultrasound examination was carried out by two deputy chief ultrasound physicians to measure the left atrial appendage volume of the patients every week, 3 times each time, and the average value was taken.

### Follow-up

The patients were followed up 12 months after successful radiofrequency catheter ablation of atrial fibrillation to observe and record the symptoms of palpitation and chest tightness. If such symptoms occurred, they were immediately sent to the hospital for a 12-lead ECG and 24-h dynamic ECG examination. The pattern of atrial fibrillation in ECG indicated a sign of recurrence.

### Observation indexes

All the data in this study were analyzed by SPSS22.0 statistical software, numerical data were expressed by number (%) and compared by $x$^2^-test. Normally distributed data was expressed by mean ± standard deviation, and independent sample t-test was used to compare the mean between the two groups. ROC curve was used to analyze the predictive value of recurrence after radiofrequency catheter ablation of atrial fibrillation. The event-free survival rate was analyzed by Kaplan–Meier estimator, and the survival difference between groups was compared by log-rank test. A *p* < 0.05 represented significant difference.

## Results

### Comparison of general data in the two groups

Age, gender, and history of diabetes were comparable between the two groups (P > 0.05), as shown in Table 1.

**Table 1:** Comparison of clinical efficacy between the two groups

Group	Recurrence group (n = 41)	Non-recurrence group (n = 155)	$x$^2^/t value	P value
Case number (male/female)	27/14	109/46	0.093	0.761
Age (year)	55.28 ± 4.01	55.87 ± 3.98	− 0.784	0.434
BMI (kg/m^2^)	25.67 ± 3.87	25.32 ± 3.28	0.534	0.594
Creatine (mmol/L)	72.89 ± 19.89	74.52 ± 13.29	− 0.496	0.622
Cardiac troponin I	7.59 ± 3.27	7.87 ± 3.71	− 0.44	0.66
C-reactive protein	2.41 ± 0.98	2.14 ± 0.26	− 1.745	0.083
Blood urea nitrogen	4.76 ± 0.82	4.45 ± 1.46	1.785	0.077
Uric acid (umol/L)	205.98 ± 49.87	221.95 ± 39.87	− 1.896	0.063
Amino terminal B type Pro-BNP (ng/L)	320.87 ± 92.38	289.67 ± 89.72	− 1.934	0.055
History of diabetes (n,%)	1 (2.44)	16 (10.32)	2.328	0.169
History of hypertension (n,%)	11 (26.83)	59 (38.06)	1.917	0.166
History of coronary heart disease (n,%)	13 (31.71)	37 (25.16)	0.697	0.404
History of heart failure (n,%)	6 (14.63)	9 (5.81)	2.946	0.086
Stroke /TIA (n, %)	2 (4.88)	11 (7.10)	0.169	0.681
ARB/ACEI (n, %)	23 (56.10)	69 (50.12)	1.746	0.186
β blocker (n, %)	12 (29.27)	29 (18.71)	2.185	0.139
Amiodarone (n, %)	9 (21.95)	16 (10.32)	3.94	0.064
Propafenone (n, %)	1 (0.02)	5 (3.23)	0.068	0.795
Characteristics of radiofrequency ablation (n, %)				
Additional linear ablation (mm)	12 (29.27)29	34 (21.94)	0.97	0.325
Additional CFAE ablation	6 (14.63)35	13 (8.39)	0.931	0.335

BMI, body mass index; TIA, transient ischemic attack; ARB/ACEI, angiotensin II receptor antagonist/angiotensin converting enzyme inhibitor

### Comparison of P-wave related electrical indexes between the two groups

P-min, PWD, P-max, PtfV1 ≥ 0.04 mV s, PR interval prolongation, and the ratio of first and third-degree IAB in the recurrence group were higher than those in the non-recurrence group, and the difference was statistically significant (Table 2).

**Table 2:** Comparison of P-wave related electrical indexes between two groups ($\overline{x} \pm s$)

	Recurrence group (n = 41)	Non-recurrence group (n = 155)	t value	P value
P-min (ms)	74.82 ± 15.87	60.18 ± 18.78	4.328	< 0.001
PWD (ms)	128.31 ± 10.77	110.03 ± 8.13	10.131	< 0.001
P-max (ms)	119.02 ± 14.27	106.99 ± 21.73	3.753	< 0.001
PtfV1 ≥ 0.04 mV·s (%)	17	17	21.03	< 0.001
PR interval	189.03 ± 21.93	175.43 ± 28.93	3.386	0.002
1st IAB, n (%)	28 (68.29)	8 (5.16)	87.281	< 0.001
3rd IAB, n (%)	6 (14.63)	5 (3.23)	7.966	0.012

**Table 3:** Comparison of cardiac image features between two groups ($\overline{x} \pm s$)

	Recurrence group (n = 41)	Non-recurrence group (n = 155)	t value	P value
Left atrial appendage volume (mL)	11.49 ± 3.86	8.26 ± 2.99	4.743	< 0.001
Left atrial volume (mL)	106.38 ± 27.83	95.87 ± 22.38	2.123	0.038
Diameter of left atrial appendage (mm)	43.47 ± 9.98	42.65 ± 8.89	0.471	0.639
Left atrial volume volume (mL)	93.02 ± 32.09	88.79 ± 28.08	0.762	0.447
Left ventricular ejection fraction (%)	52.38 ± 7.98	53.93 ± 7.56	− 1.067	0.288

### ROC curve of PWD, P-max, PtfV1 and prediction of recurrence

**Fig. 6:**
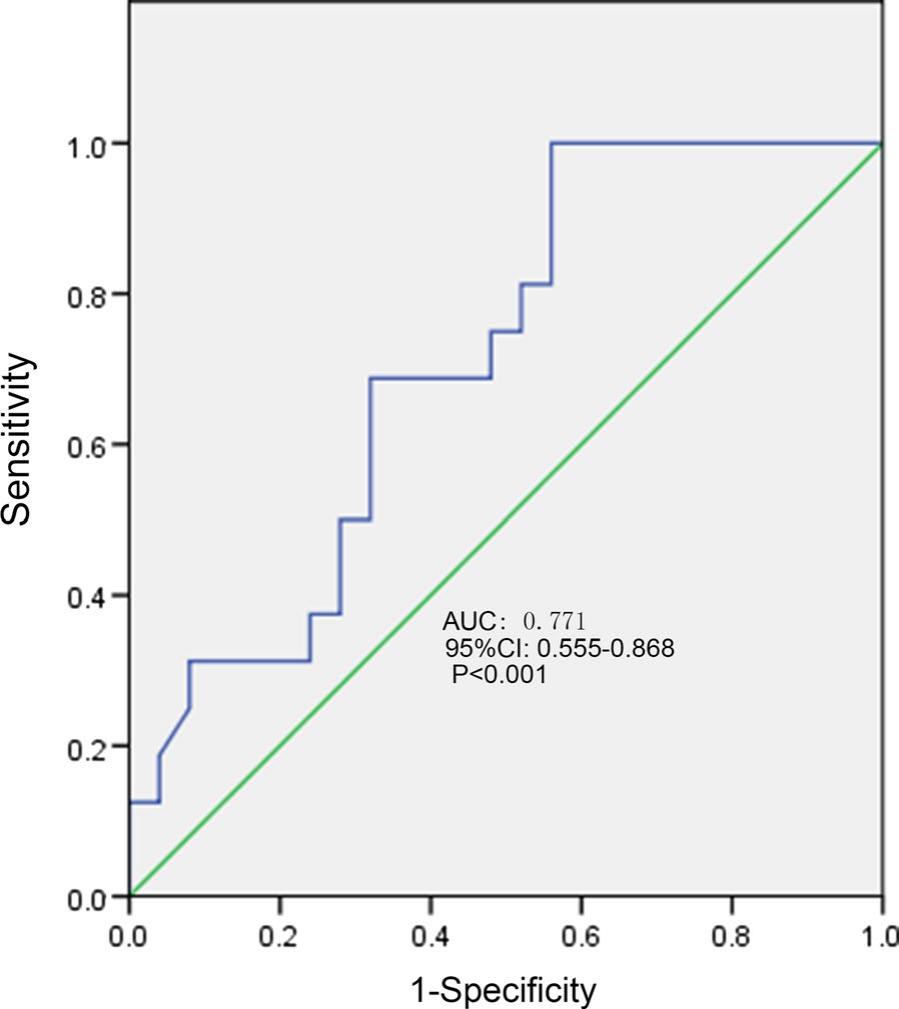
ROC curve of P wave dispersion predicting the recurrence of atrial fibrillation after catheter ablation

**Fig. 7:**
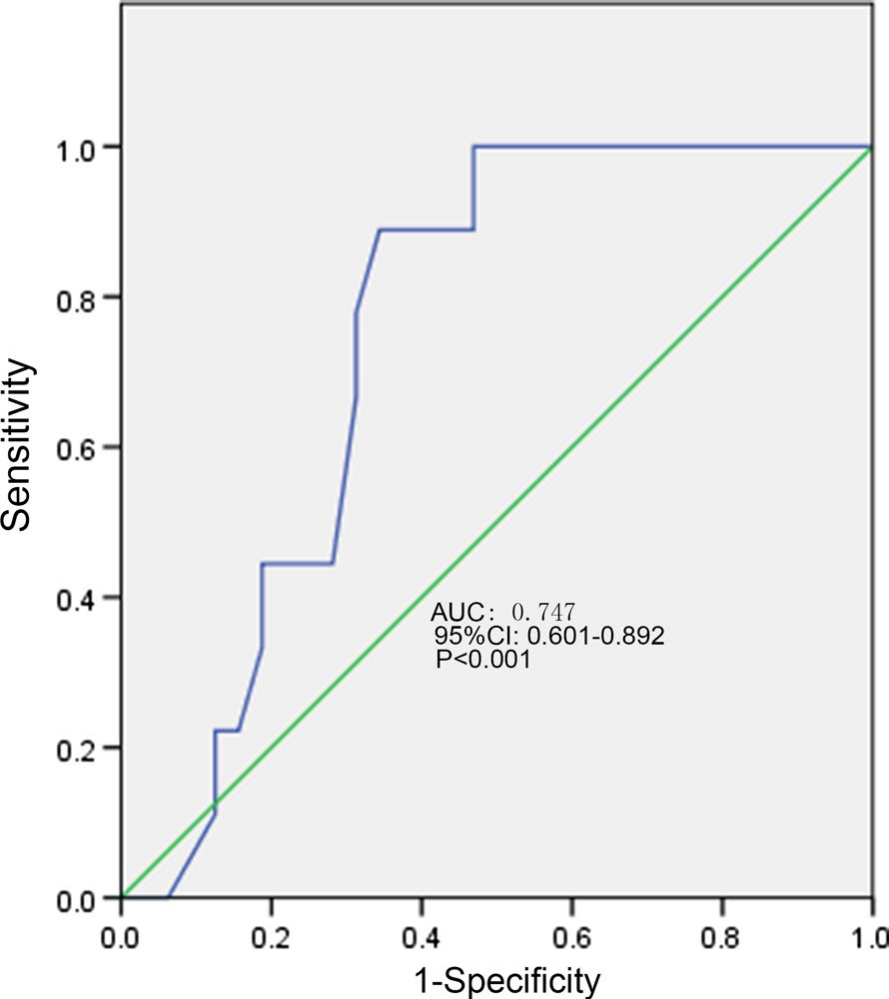
ROC curve of maximum P-wave duration predicting the recurrence of atrial fibrillation after catheter ablation

**Fig. 8:**
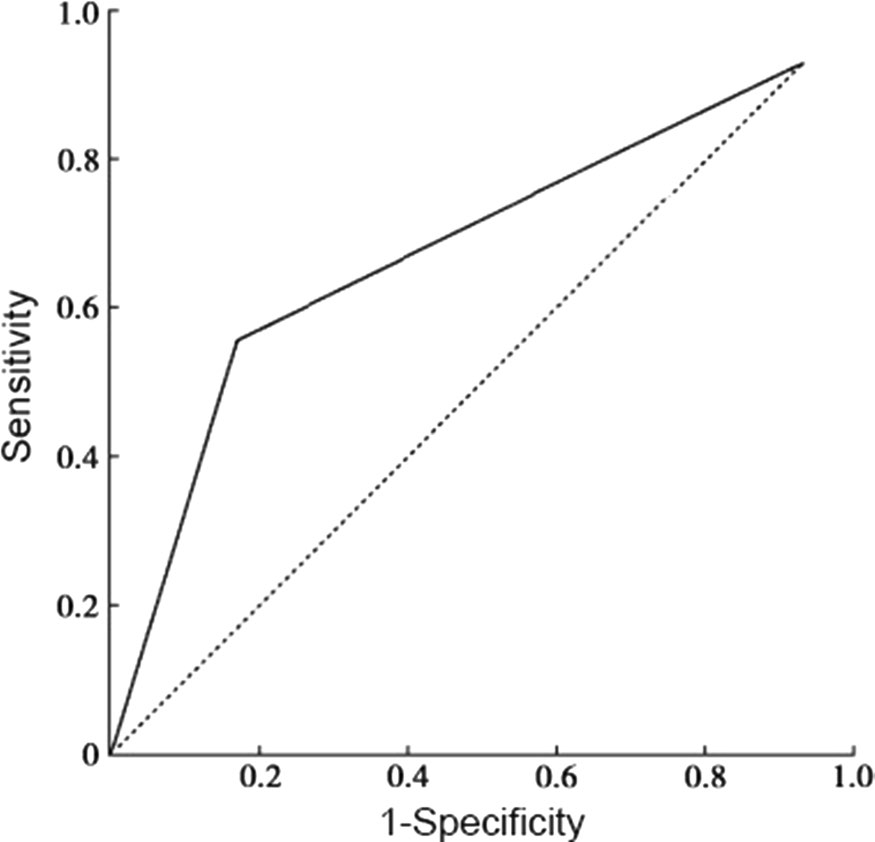
ROC curve of V1 lead P-wave terminal potential predicting the recurrence of atrial fibrillation after catheter ablation

**Fig. 9:**
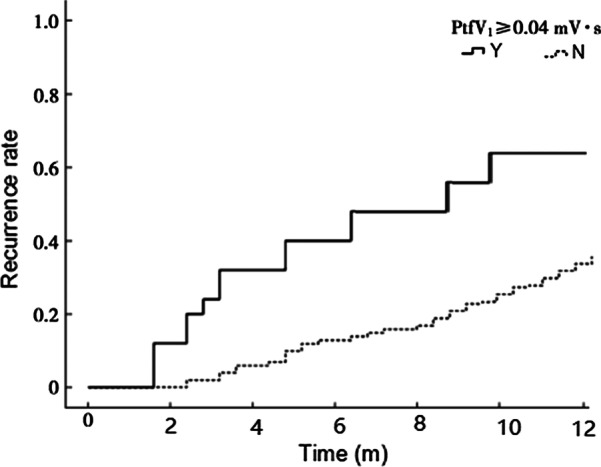
Kaplan–Meier curve analysis of the time to recurrence of atrial fibrillation after catheter ablation when PtfV1 ≥ 0.04 mV·s

The ROC curves of PWD, P-max, and PtfV1 are shown in Figs. 6, 7 and 8. Further analysis showed that when PWD was 105.6 ms, the sensitivity, specificity, positive predictive value, negative predictive value (Table 3), and accuracy of atrial fibrillation recurrence after catheter ablation were 74.18%, 71.9%, 41.27%, 90.89%, and 72.76%, respectively. When P-max was 117.3, the sensitivity, specificity, positive predictive value, negative predictive value, and accuracy of atrial fibrillation recurrence after atrial fibrillation catheter ablation were 93.2%, 62.1%, 55.6%, 97.6%, and 74.2%, respectively. Kaplan–Meier curve analysis was performed on time to atrial fibrillation recurrence after atrial fibrillation catheter ablation when PtfV1 ≥ 0.04 mv s by comparison between groups (Log rank test: 2 = 4.739, P < 0.001) (Fig. 9). When PtfV1 was ≥ 0.04 mV s, the sensitivity, specificity, positive predictive value, negative predictive value, and accuracy of atrial fibrillation recurrence after atrial fibrillation catheter ablation were 72.31%, 74.03%, 59.08%, 98.01%, and 73.29%, respectively (“See ROC data in Additional file 1”).

### Comparison of cardiac image features between the two groups

In the CT image features of the two groups, the left atrial appendage volume and left atrial volume in the recurrence group were significantly higher than those in the non-recurrence group (P < 0.05). Based on the echocardiographic features, the diameter of the left atrial appendage and left atrial volume in the recurrence group were higher than those in the non-recurrence group, but without significant difference (P > 0.05), and there was no significant difference in the left ventricular ejection fraction between the two groups (P > 0.05).

### Left atrial appendage volume and prediction of recurrence of atrial fibrillation

**Fig. 10:**
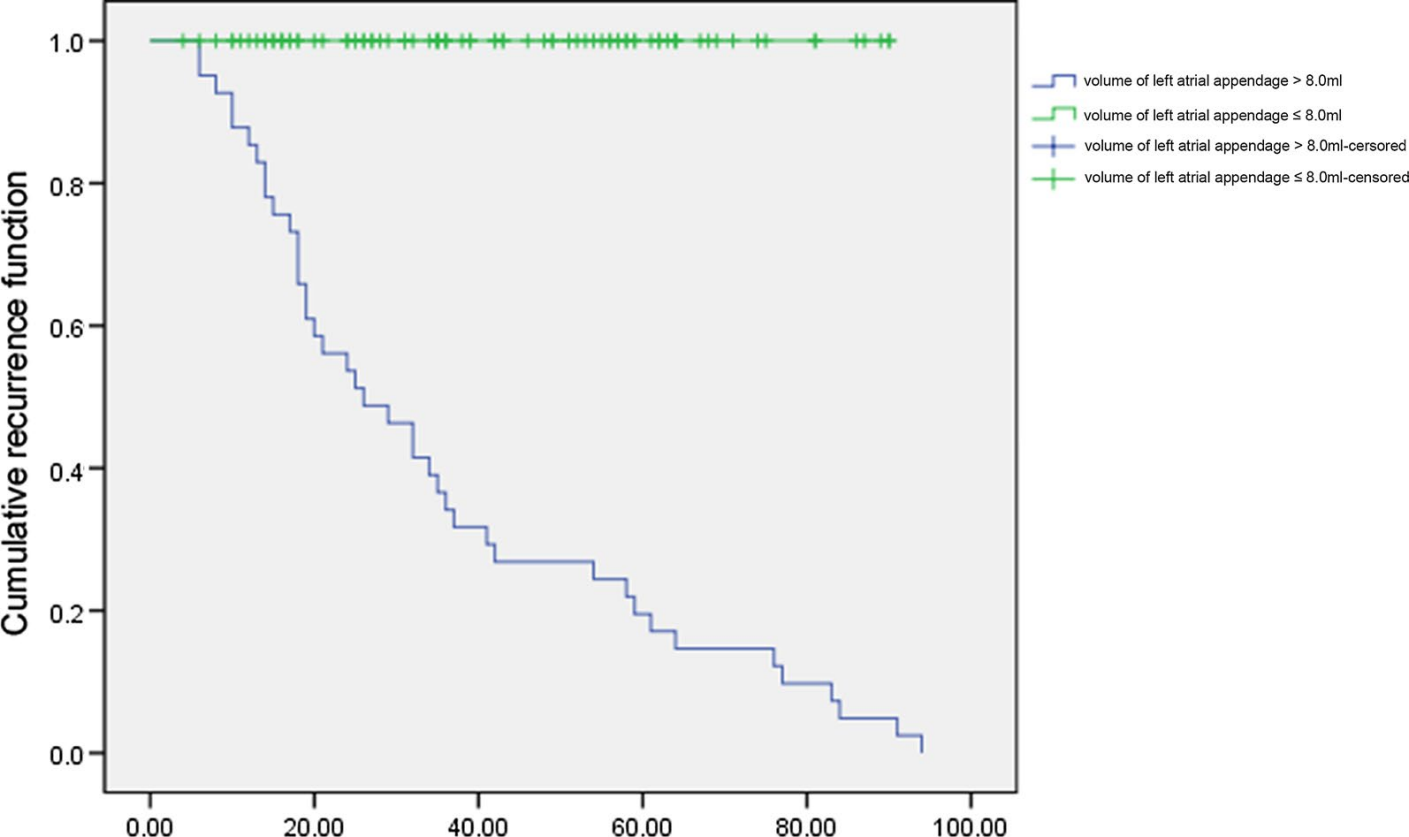
Kaplan–Meier curve analysis of the left atrial appendage volume and AF-free survival after AF ablation

When the volume of the left atrial appendage was > 8.0 mL, the sensitivity, specificity, positive predictive value, negative predictive value, and accuracy of atrial fibrillation recurrence after atrial fibrillation catheter ablation were 95.12%, 70.73%, 76.47%, 93.55%, and 82.93%, respectively. Kaplan–Meier curve analysis showed that the survival rate without recurrence of atrial fibrillation after catheter ablation was lower when the volume of the left atrial appendage exceeded 8.0 mL (log-rank test P < 0.001, Fig. 10).

## Discussion

Currently, the main clinical treatment of atrial fibrillation is catheter radiofrequency ablation, which has been widely focused on and recommended by the guidelines with a success rate as high as 80% [5, 6]. Nevertheless, it has a high recurrence rate. Some patients need to undergo surgery for one or several times, which has a negative impact on health and inflicts an economic burden on patients. In this study, 196 patients with paroxysmal atrial fibrillation were followed up for more than one year. The success rate of transcatheter radiofrequency ablation was 79.08%, and the recurrence rate was 20.92%. Among them, 5 patients underwent catheter ablation again, without recurrence after the operation, which is similar to those of previous studies. Several previous studies [7] investigated the risk factors of recurrence of atrial fibrillation, including inflammatory factors, age, left atrial scar and atrial fibrosis, duration of atrial fibrillation, overweight and obesity, organic heart disease, and so on. At present, there are limited studies on the effects of P-wave ECG index and the left atrial appendage volume on the recurrence of paroxysmal atrial fibrillation treated by radiofrequency catheter ablation [8, 9].

P-min, PWD, P-max, PtfV1, PR interval prolongation, and IAB are ECG indexes related to P-wave, which may be linked to atrial fibrillation and its recurrence. Among the abovementioned indexes, P-min and PWD reflect discontinuous conduction and heterogeneity in the atrium. At present, numerous clinical studies were published on PWD in various fields. In addition, the most useful clinical value of PWD includes the prediction of atrial fibrillation in patients with coronary artery bypass grafting, coronary heart disease, hypertension and no obvious heart disease. Related studies are available to show that PWD is the most sensitive muscle-specific factor in ECG prediction of atrial fibrillation [10], and PWD also has a potential value in recurrence after radiofrequency catheter ablation [11].

The PR interval represents the time for electrical activation to pass from the atrial muscle near the sinus node to Purkinje fibers through the atrioventricular node. The prolongation of the PR interval is due to delay in atrioventricular node conduction, which is usually defined as the PR interval > 200 ms. Therefore, it is generally believed that for patients without organic heart disease, the prolongation of the PR interval is only a benign change. However, recent studies have suggested that there is a certain correlation between the prolongation of the PR interval and the occurrence and progression of atrial fibrillation. A recent literature review found that PR interval prolongation is related to AF [12]. The genetic background of PR interval prolongation is similar to that of atrial fibrillation. Autonomic nerve regulation can affect the PR interval, and abnormal cardiac autonomic nerve function can be manifested as a prolonged PR interval. Atrial electrical remodeling and structural remodeling can be manifested as a prolonged PR interval. The above factors are important in the occurrence and progression of atrial fibrillation. However, there are few studies on the correlation between PR interval and postoperative AF recurrence.

Based on the results of this study, P-min, PWD, P-max, PR interval prolongation, and the ratio of first and third-degree IAB in the recurrence group were higher than those in the non-recurrence group (p < 0.05). The ROC curve showed that PWD was 0.771 and P-max was 0.747, suggesting that P-min, PWD, P-max, PR interval prolongation, and first and third-degree IAB might have great predictive value for atrial fibrillation recurrence after catheter ablation. Besides, when PWD was 105.6 ms, the sensitivity, specificity, positive predictive value, negative predictive value, and accuracy of atrial fibrillation recurrence after catheter ablation were 74.18%, 71.9%, 41.27%, 90.89%, and 72.76%, respectively. When P-max was 117.3, the sensitivity, specificity, positive predictive value, negative predictive value, and accuracy of atrial fibrillation recurrence after catheter ablation were 93.2%, 62.1%, 55.6%, 97.6%, and 74.2%, respectively. The results indicated that PWD of 105.6 ms and PWD max > 117.5 ms were of high value in predicting recurrence after catheter ablation.

Salah et al. [13] found that P wave duration > 125 ms, P wave dispersion > 40 ms and PWTF in V1 < − 0.04 mm/s are good clinical predictors of AF recurrences post PVI in patients with paroxysmal atrial fibrillation. IAB was proposed by Bayés et al. [14] according to the length of PWD and the direction of P wave in leads II, III and aVF. Enriquez et al. [15] indicated that the ratio of first-degree IAB and third-degree IAB in the AF recurrence group were significantly higher than those in the non-recurrence group, suggesting that the presence of third-degree IAB is an independent risk factor for atrial fibrillation recurrence. The reason is that the Bachmann's bundle is blocked in third degree IAB, and the impulse cannot be transmitted to the left atrium, which passes through the myocardial connection near the coronary sinus and activates the left atrium retrogradely, leading to delayed activation of the left atrium, synchronization of the left and right atria, increase in the conduction heterogeneity of the left atrium, and impairment of the systolic function of the left atrium. The above factors can cause AF recurrence.

The anterior initial vector of P-wave in ECG is right atrial activation, and the posterior terminal vector is left atrial activation; hence, it is called left and right atrial comprehensive depolarization wave. PtfV1 represents the area of transverse left atrial depolarization vector, and the increase of PtfV1 represents larger left atrial depolarization vector, indicating abnormal left atrial interatrial conduction [16]. PtfV1 abnormalities can be caused by an enlarged left atrium, backward transfer of the left atrium, limitation of left atrial conduction velocity, and left atrial hypertrophy due to hemodynamic pressure. According to the study by Martín et al. [17], the OR value of recurrent atrial fibrillation was 1.9 when the negative terminal of lead V1 decreased more than 0.1 mV and maintained 40 m. In order to avoid the error caused by ECG itself or the small value of PtfV1, PtfV1 ≥ 0.04 mV·s was set as the cutoff value in the present study, and its measurement was convenient with good analytic effect. The area under the ROC curve was measured based on PtfV1 ≥ 0.04 mV·s by ROC curve analysis of predictive ability, suggesting a high value of predicting recurrence after catheter ablation of atrial fibrillation. The results of this study suggested that the sensitivity, specificity, positive predictive value, negative predictive value, and accuracy of PtfV1 ≥ 0.04 mV·s for recurrence of atrial fibrillation after catheter ablation were 72.31%, 74.03%, 59.08%, 98.01%, and 73.29%, respectively. It can be inferred that when the absolute value of PtfV1 after catheter ablation is large, the risk of atrial fibrillation recurrence increases. PtfV1 can be used as a new non-invasive examination index to evaluate the recurrence of atrial fibrillation after catheter ablation, and the cutoff value of PtfV1 ≥ 0.04 mV ·s showed greater beneficial value for clinical practice.

The left atrial appendage is namely an appendage of the left atrium, and there are complex and staggered myocardial fibers at the junction. The anisotropic myocardial fibers at the junction can accelerate atrial beats at this site, resulting in abnormal electrophysiological characteristics [18, 19]. In addition, the comb muscle in the left atrial appendage can delay and prevent electrical signal transmission and lead to atrial arrhythmia; hence, the left atrial appendage can trigger atrial fibrillation. According to related studies [20, 21], hypertension, organic heart disease, the duration of atrial fibrillation, the anterior and posterior diameter of the left atrium and left ventricular ejection fraction are related to the recurrence of atrial fibrillation after ablation and have an association with the occurrence and maintenance of atrial fibrillation matrix. Morever, the volume of the left atrial appendage and the number of lobes are significantly related to the remodeling of the left atrial structure in atrial fibrillation, which may lead to enhanced autonomy and multiple reentries and spiral reentry in the local atrial anatomy, inducing recurrence of atrial fibrillation. Di et al. [22] summarized and analyzed 987 patients with failed ablation of atrial fibrillation in 2010, demonstrating that the left atrial appendage was related to the recurrence of atrial fibrillation. Another study followed up patients with isolated atrial fibrillation after ablation for 5 years, suggesting that the anterior and posterior diameter of the left atrium is related to the recurrence of atrial fibrillation after radiofrequency ablation [23]. Pinto et al. [9] also confirmed that the left atrial volume is an independent risk factor for recurrence of atrial fibrillation. Based on the results of our study, the volume of the left atrial appendage and the left atrium in the recurrence group were higher than those in the non-recurrence group in the CT image features of the two groups (P < 0.05). The echocardiographic features showed that the diameter of the left atrial appendage and left atrial volume in the recurrence group were higher than those in the non-recurrence group, but without significant difference (P > 0.05), and there was no significant difference in the left ventricular ejection fraction between the two groups (P > 0.05). The results suggested that both the left atrial volume and the left atrial appendage volume can be used as effective indicators of atrial arrhythmias, and the measurement data of the left atrial appendage volume by cardiac enhanced CT before the operation were considered to be accurate and feasible. In the present study, the volume of left atrial appendage and the prediction of recurrence of atrial fibrillation were analyzed. When the volume of the left atrial appendage exceeded 8.0 mL, the sensitivity, specificity, positive predictive value, negative predictive value, and accuracy of atrial fibrillation recurrence after catheter ablation were 93.7%, 66.21%, 58.72%, 93.76%, and 75.72%, respectively. Kaplan–Meier curve analysis showed that the survival rate without recurrence of atrial fibrillation after catheter ablation was lower when the volume of left atrial appendage exceeded 8.0 mL (log-rank test P < 0.001), indicating that the preoperative measurement of the left atrial appendage volume can identify patients at high risk of recurrence after radiofrequency ablation in advance. Therefore, the left atrial appendage volume can be used as an effective factor to predict the recurrence of atrial fibrillation after radiofrequency ablation.

## Conclusion

PWD, P-max, PtfV1, PR interval prolongation, first and third-degree IAB, and the left atrial appendage volume are considered to be effective predictors of recurrence after first radiofrequency catheter ablation. Admittedly, some limitations should be acknowledged. During the follow-up, outpatient follow-up twice a week, ordinary ECG, and dynamic ECG once a week were performed to detect the recurrence of atrial fibrillation, while a small number of asymptomatic patients with atrial fibrillation could not be detected in time or ignored. Besides, given the limited time and sample size of our study, further studies are warranted to confirm the current findings (Additional file 1).

## Supplementary information

**Additional file 1:** ROC data for PWD, P-max, PtfV1 and prediction of recurrence.

## Data Availability

The datasets used and/or analysed during the current study are available from the corresponding author on reasonable request.

## Abbreviations

LLAV: Left atrial appendage volume
mPWD: Mean P-wave duration
P-max: Maximum P-wave duration
P-min: Minimum P-wave duration
PtfV1: P-wave terminal force in lead V1
PWD: P-wave dispersion
